# Eight rules for the haemodynamic management of traumatic brain-injured patients

**DOI:** 10.1097/EA9.0000000000000029

**Published:** 2023-06-22

**Authors:** Simone Di Filippo, Antonio Messina, Paolo Pelosi, Chiara Robba

**Affiliations:** From the Department of Biotechnology and Sciences of Life, Anesthesia and Intensive Care, ASST Sette Laghi, University of Insubria, Varese (SDF), IRCCS Humanitas Research Hospital, Via Alessandro Manzoni, Rozzano (AM), Department of Biomedical Sciences, Humanitas University, Pieve Emanuele, Milan (AM), IRCCS Ospedale Policlinico San Martino (PP, CR) and Department of Surgical Sciences and Integrated Diagnostics, DISC, University of Genoa, Genoa, Italy (PP, CR)

## Abstract

Traumatic brain injury (TBI), a leading cause of death and poor neurological outcomes in trauma patients, is a primary cause of severe disability among survivors and a major public health burden globally. Optimal haemodynamic management is a keystone of care in avoiding secondary brain injury, and contributes to minimising mortality and morbidity. Although some important progress has been achieved, a paucity of high-quality recommendations still exists. The purpose of this article is to review the current knowledge on TBI-associated haemodynamic tenets, in order to summarise the most important aspects of this heterogeneous and complex field.

**KEY POINTS**

- A reciprocal ‘crosstalk’ between the cardiovascular and the cerebrovascular systems exists.
- Cardiological complications after acute brain injury are common and significantly influence patients’ outcome.
- Coagulation disorders can influence the progression of intracranial haemorrhage and increase the risk of mortality and poor neurological outcomes.
- Fluid balance and criteria for haemodynamic management are based on low level of evidence and the haemodynamic treatment of these patients should be individualised.

## Introduction

Annually, traumatic brain injury (TBI) affects more than 60 million patients worldwide^1^ and remains one of the leading causes of mortality across all ages.^2,3^ Due to its complex pathophysiology, a large proportion of patients will suffer secondary brain damage and long-term disability, despite improvements in critical care management.^2,4^ To reduce the risks of developing secondary injury, comprehensive haemodynamic management (including monitoring, fluids and vasopressor therapy) is pivotal for preserving cerebral perfusion pressure (CPP) and ensuring an adequate cerebral blood flow (CBF) and oxygenation. Both hypotension and hypertension are detrimental for brain health, especially after a traumatic event. Moreover, acute cardiac dysfunction, because of brain–heart crosstalk, and trauma-related coagulopathy may further worsen the patient's clinical status.^5^ During the last decades, several recommendations have been proposed for the haemodynamic management of TBI patients but, state-of-the-art, strong evidence is limited.^6,7^ This is reflected in a large variation in the management of critically ill patients with TBI. With this review article, we aim to give a comprehensive insight into TBI-associated haemodynamic aspects in order to summarise the most important aspects of this heterogeneous and complex field.

## Brain–heart crosstalk really exists

**Fig. 1:**
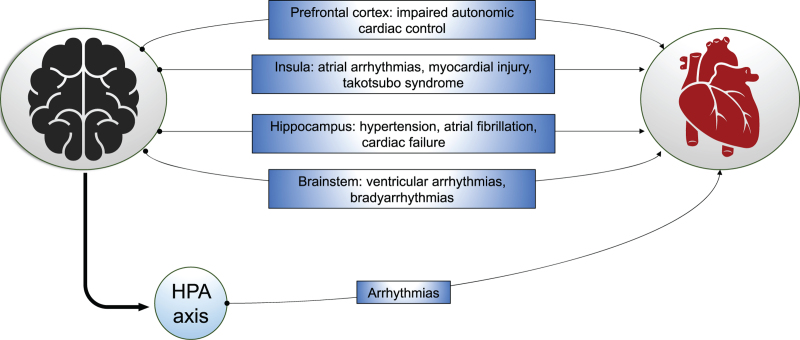
Representation of brain–heart crosstalk. Different areas of the brain can, when damaged, cause cardiological alterations resulting in different clinical pictures. For instance, a brain injury localised in the prefrontal cortex can lead to impaired autonomic cardiac control, whereas brainstem lesions more often cause arrhythmias.

In recent times, interdependence between biological systems has been thoroughly studied. Interestingly, as a growing body of evidence suggests, the tight interconnection between the brain and other organs seems to be responsible for the extracerebral complications that frequently occur after TBI.^8^ Brain–heart crosstalk has been studied for decades, since the Cushing reflex was described.^9,10^ In this regard, a reciprocal crosstalk between the cardiovascular and the cerebrovascular systems, consisting of several afferent and efferent projections, was demonstrated and the study of the neurocardiac axis has become known as neurocardiology (Fig. 1).^11^ Some of these effects are similar to those described during the so-called ‘stroke-heart syndrome’, referring to a wide spectrum of cardiac dysfunction related to all the acute brain injuries^12,13^ and involving the so-called hypothalamic–pituitary–adrenal (HPA) axis, whose dysregulation may affect the cardiovascular system.^14,15^

HPA, hypothalamic–pituitary–adrenal.

The heart is capable of modulating its own rhythm and contractility. However, an xtensive network of cortical and subcortical projections, forming the autonomic nervous system (ANS), and coordinated by the central nervous system (CNS) also affect cardiac function in response to a plethora of inputs.^16^ In particular, the ANS consists of sympathetic and parasympathetic fibres networking with the intrinsic cardiac nervous system and influencing myocardial activity,^16,17^ with several brain structures involved (e.g. amygdala, hippocampus, hypothalamus, cortex and brain stem areas). Within this complex network, the insula seems to play a crucial role in adjusting cardiovascular function either via sympathetic or parasympathetic responses.^18,19^

Extracerebral complications frequently occur after TBI and significantly affect patient outcome.^8,20^ Brain–heart crosstalk seems to be a crucial actor in the pathogenesis of cardiac dysfunction. Along with pulmonary complications, acute cardiac impairment represents the most common extracranial consequence in TBI patients.^8^ It is well known that the cardiovascular system is able to affect the CNS (such as hypoperfusion, oedema and embolic stroke). Reciprocally, cerebrovascular accidents may cause several acute cardiac dysfunctions such as cardiac enzyme release, arrhythmias, ischaemia and, in the worst scenario, cardiac failure.^21–23^ According to the most widely accepted theory, most of these changes seem to be related to an abnormal catecholamine exposure. The sympathetic surge may cause a massive release of epinephrine and norepinephrine from sympathetic projections leading to stress-related cardiomyopathies, which may impair cardiac function in different ways, including heart rate variability, baroreceptor reflex sensitivity, intracardiac conduction and variable degrees of acute myocardial dysfunction.^24,25^ In cases with cardiac failure, the haemodynamic state may be insufficient to sustain cerebral metabolic demand, leading to the neurological status worsening and unfavourable outcomes. Hence, once any primary causes for pathological cardiovascular states have been excluded, extracardiac sources of myocardial abnormalities should be always taken into consideration.^18^

## Coagulopathies are common after traumatic brain injury

**Fig. 2:**
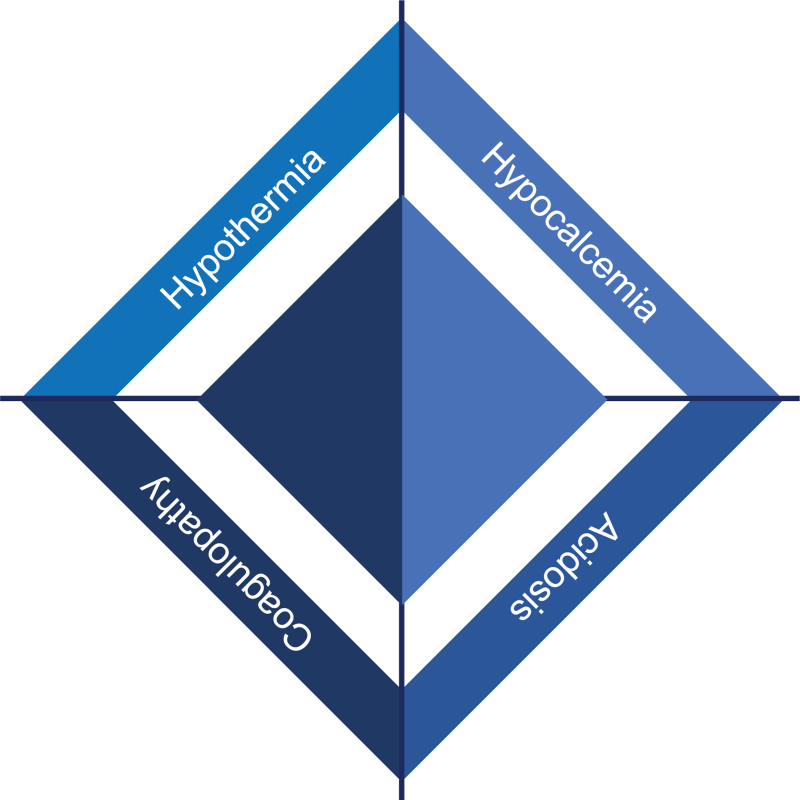
The classical lethal triad (acidosis, coagulopathy, hypothermia) illustrated as the diamond of death. The negative effects of hypocalcaemia are intrinsically linked to components of the lethal triad having a direct and indirect effect on each portion of the lethal triad, supporting calcium's potential position as a fourth component in this lethal diamond.

Up to several hours after TBI, a significant proportion of patients develop an acute trauma-induced coagulopathy (TIC) potentially resulting in haemorrhagic progression of an intracerebral contusion and worsening of the secondary brain injury.^26^ According to current literature, TIC prevalence is estimated to range between 27 and 35.2% and is associated with higher blood transfusion rates, prolonged hospitalisation, delayed weaning from ventilation and increased risk of multiple organ failure.^27–29^ Although TIC is associated with a high mortality rate and poor prognosis, its underlying mechanisms have not been fully explained.^26,29^ Extracranial TIC is often multifactorial with causal factors both endogenous (e.g. haemorrhagic shock) and exogenous (e.g. haemodilution).^30^ However, TBI-associated coagulopathy lacks these features suggesting that the pathogenesis may be because of other aetiologies.^31^ Recently, it was postulated that blood–brain barrier (BBB) disruption may lead to a massive release of specific molecules triggering the coagulopathy.^31^ In particular, subendothelial tissue factor, which is abundant in the brain, and several pro-inflammatory cytokines may be involved in the indiscriminate activation of the coagulation system.^28,31^ The systemic activation of the coagulation cascade may progress to disseminated intravascular coagulation with subsequent haemorrhagic diathesis because of clotting factor consumption.^32,33^ Lastly, the coagulation dysfunction may be further complicated by co-existing acidosis, hypothermia and hypocalcaemia in what is called the ‘diamond of death’ (Fig. 2).^34^

TIC, resulting from the sum of haemorrhage, haemodilution, acidosis, hypothermia, hypocalcaemia, fibrinolysis and clotting factor consumption, demands early recognition and goal-directed treatment upon hospital admission.^28^ Unfortunately, specific guidelines for the treatment of TIC in head-injured patients do not exist and its management varies widely amongst centres. According to the European guidelines on the management of major bleeding and coagulopathy following trauma,^35^ coagulation monitoring using standard laboratory-based tests, such as platelet count, prothrombin time (PT), international normalised ratio (INR), activated partial thromboplastin time (aPTT) and fibrinogen levels is recommended, despite their considerable turnaround time. In contrast, viscoelastic tests, such as thromboelastography (TEG) and rotational thromboelastometry (ROTEM), are able to deliver the result within a few minutes and provide helpful information on clot strength and fibrinolysis.^36,37^

Platelet dysfunction parallels coagulopathy after trauma. Consequently, thrombocyte function assays may provide further relevant clues, although it is not always feasible in an emergency setting.^38^ Moreover, the red blood cell count should be taken into account when managing TIC because an adequate haematocrit, affecting blood viscosity and platelet adhesion, is required to promote blood coagulation.^39^ An additional informative bedside test is arterial blood gas analysis used to quickly rule out or treat acidosis and hypocalcaemia, two well known TIC-precipitating factors. Finally, measurement and treatment of core body temperature should be undertaken to decrease the risk of TIC during trauma resuscitation.^40,41^

Goal-directed therapy of haemostatic abnormalities is a priority in TBI patients. Several options to limit the effects of coagulopathy are available, depending on individual requirements. In detail, allogeneic transfusion of packed red blood cells should be considered to target a haemoglobin concentration between 7 and 9 g dl^−1^, thus avoiding poor oxygen delivery.^42^ Platelet concentrates should be administered to maintain thrombocythaemia above the optimal count for TBI patients (>100 000 μl^−1^).^43^ Similarly, fresh frozen plasma should be administered to replace clotting factor loss, including fibrinogen, to increase the haemostatic potential. Fibrinogen, also known as factor *I*, is the first coagulation factor to decrease during blood loss. Low levels of factor *I* (<200 mg dl^−1^) at hospital admission are correlated with higher mortality in trauma patients. Hence, when hypofibrinogenemia is detected by TEG or ROTEM analysis, purified human fibrinogen supplementation is recommended at an initial dose of 3 to 4 g.^44^

Another useful weapon against TIC is tranexamic acid (TXA), a synthetic antifibrinolytic agent. The CRASH-3 randomised trial demonstrated that TXA may have a role in reducing haemorrhage expansion, brain herniation and death in mild-to-moderate TBI patients. TXA should be infused intravenously within 3 h of the traumatic event at a loading dose of 1 g over 10 min, followed by a maintenance dose of 1 g over 8 h.^45^

Patients taking antithrombotic drugs (antiplatelet agents, vitamin K-dependent anticoagulants and direct oral anticoagulants) may benefit from emergency reversal. Further platelet administration is indicated in TBI patients who received antiplatelet agents, whereas prothrombin complex concentrates (PCC) should be administered in association with vitamin K to counteract vitamin K-dependent clotting factor loss (II, VII, IX, X). For patients treated with direct oral anticoagulants, PCC can be safely administered, especially when no specific antidotes are available.^46^ On the other hand, recombinant-activated coagulation factor VII is contraindicated as a first-line treatment because of the increased risk of thromboembolic events.^35,47^

## Arterial blood pressure targets after traumatic brain injury: avoid blood pressure instability

Among the numerous TBI-associated complications, blood pressure alterations and haemodynamic instability play a major role in causing secondary brain injury.^48^ Both low and high systolic blood pressure (SBP) can be detrimental, especially for the injured brain.^49^ Hence, preventing or minimising pressure fluctuations is a cornerstone in TBI management.^50^ In this regard, it has been shown that early hypotension, that mostly originates from associated injuries such as myocardial contusion, extracerebral bleeding and secondary polyuria, considerably increases mortality after TBI because of cerebral perfusion compromise.^51,52^ For this reason, the latest edition of the Brain Trauma Foundation (BTF) guidelines suggest a prompt, aggressive hypotension reversal, targeting SBP greater than 100 to 110 mmHg specifically to support CPP and to improve neurological outcome.^6^ Judicious crystalloid administration is the first-line therapy to achieve euvolaemia in these patients. In fact, inappropriate fluid administration may lead to pulmonary oedema and worsen cerebral swelling. In ‘fluid nonresponder’ patients, vasoactive agents, such as norepinephrine or phenylephrine, are recommended.

**Fig. 3:**
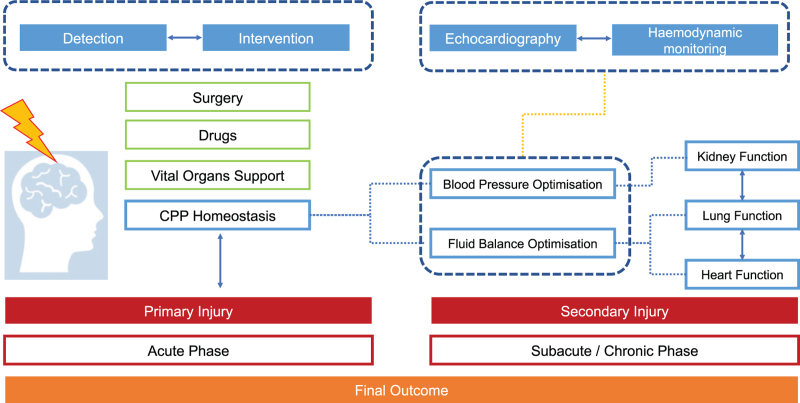
Schematic representation of haemodynamic management of patients with traumatic brain injury. The patient's outcome is linked to the combination of primary and secondary injuries. Haemodynamic optimisation aims at pressure and fluid balance improvement, maintaining appropriate cerebral perfusion and preventing secondary damage of other organs, such as kidneys, lungs and heart. Critical care echocardiography and haemodynamic monitoring may help in titrating therapies, and should be considered for complex patients.

On the other hand, hypertension prevents mean arterial pressure (MAP) decrease and may be neuroprotective at first. However, sustained catecholamine release may lead to secondary brain injury by increasing intracranial pressure (ICP) and promoting cerebral oedema. Current guidelines do not set an optimal pressure threshold in these patients.^53^ Lastly, it has been shown that blood pressure fluctuations after TBI are strongly associated with haematoma progression and worse outcomes, underlining the importance of a tight SBP control in these patients. Wherever cerebral autoregulation is impaired, the underlying factors must be sought out.^54^ The combined use of multimodal monitoring, such as cardiac output (*CO*) and ICP, may be effective to enable the required interventions, optimising fluid administration and vasoactive therapy (Fig. 3).^55^

## Vasopressors and inotropes in traumatic brain injury

The BTF guidelines recommend targeting a cerebral perfusion pressure value between 60 and 70 mmHg, with a level IIb evidence for survival and favourable outcomes.^6,56^ However, an optimal arterial blood pressure (ABP) should also target an adequate CPP when autoregulation is lost.^57^ Massive fluid administration can lead to life-threatening complications. Hence, in current clinical practice, vasoactive agents are extensively employed as useful fluid-sparing adjuncts. Vasopressors (phenylephrine, ephedrine, vasopressin) augment systemic vascular resistance (SVR) via vasoconstrictive adrenergic or vasopressin receptors, and inotropes (dopamine, norepinephrine, epinephrine) increase cardiac contractility (with variable effects on SVR). The result is a marked rise in MAP and *CO* improvement.^58^ However, because of BBB disruption, the protective effect of monoamine oxidases, which act as a biochemical barrier metabolising endogenous and exogenous amines, is prone to fail. Therefore, these drugs must be wisely used in TBI patients, as excessive vasoconstriction may paradoxically worsen cerebral capillary blood flow.^59,60^

Current guidelines advocate the early use of vasopressors following TBI, although without any recommendation on the drug choice. A broad variability in clinical practice exists.^61^ Several studies found that phenylephrine and norepinephrine were the most common vasoactive drugs employed, with no significant differences in any clinical outcomes.^61–63^ At present, norepinephrine is the most commonly used vasoactive agent as first-line therapy when ABP increase is required.^64^ After restoring MAP, and following an accurate evaluation of cardiac function, inotropic support may be required to sustain *CO* and improve systemic oxygen delivery.^65^

## When to use haemodynamic monitoring in traumatic brain injury patients

A haemodynamic profile assessment is pivotal to optimise cerebral and systemic perfusion pressure in neurocritical patients. In fact, suboptimal haemodynamic management may exacerbate secondary brain injury via multiple mechanisms, such as hypotension, hypoxaemia and anaemia.^2^

In a prehospital setting, blood pressure and heart rate are normally measured to estimate blood loss and to avoid hypotension.^66^ In addition, electrocardiography and invasive monitoring of ABP are the standard of care in critical areas. However, as CBF is exceptionally sensitive to systemic blood pressure variations after TBI, more advanced monitoring techniques, both invasive and noninvasive, may be necessary.^64^ These tools are able to provide continuous, accurate and more informative data that enable a timely detection of pathological changes, ultimately improving patient's outcome (Fig. 3).^67,68^ Although, the thermodilution technique via a pulmonary artery catheter is considered the clinical gold standard to measure *CO* and other haemodynamic parameters, it is rarely employed because of its invasiveness. On the other hand, ABP-based systems, including transpulmonary thermodilution and arterial contour analysis, or totally noninvasive techniques, such as echocardiography, are being adopted increasingly. According to a recent European Society of Intensive Care Medicine (ESICM) survey,^64^ systemic haemodynamic monitoring should be considered in brain-injured patients in order to optimise treatment with regard to CBF and oxygen delivery.^69,70^

In the last decades, the use of oesophageal Doppler monitoring has been extensively validated both during surgery and in intensive care for the assessment of patients’ haemodynamic status. Thanks to its minimal invasiveness, it could facilitate optimal titration of intravenous fluids via real-time measurement of stroke volume and *CO*.^71,72^ Consequently, it has the potential to be a helpful tool in mechanically ventilated TBI patients for noninvasive ascertainment of fluid responsiveness and therapy individualisation,^73^ despite evidence lacking in the context of isolated TBI.

## Consider autoregulation: one size of target does not fit all!

CPP is the driving force that provides blood to the cerebral tissue and rigorously depends on ICP and MAP, according to the formula CPP = MAP − ICP.^74^ Prolonged and excessively low CPP represents an important and preventable cause of secondary brain injury.^57^ According to the BTF guidelines, CPP values should range between 60 and 70 mmHg in order to avoid cerebral hypoperfusion.^6^ However, a fixed threshold may not be appropriate for every patient, as it does not take into account existing interindividual variability, especially in severely brain-injured patients.^75^ Cerebrovascular autoregulation is a vital homeostatic mechanism able to maintain a constant CBF (50 ml 100 g^−1^ min^−1^) over a wide range of CPP (50 to 150 mmHg), according to Lassen's curve.^76,77^ Several mechanisms, including myogenic, neurogenic, endothelial and metabolic, often acting in combination, are involved in cerebrovascular autoregulation mediation.^78^ As long as cerebrovascular autoregulation is functional, the brain counteracts inappropriate MAP fluctuations preventing cerebral ischaemia, raised ICP or hyperaemia.^79^ However, in TBI patients, cerebrovascular autoregulation may be disrupted and the autoregulation plateau narrowed leading to inadequate CPP, exacerbating secondary brain injury.^80^ Unsurprisingly, cerebrovascular autoregulation failure is associated with a poor prognosis.^81^ Cerebrovascular autoregulation is measurable by analysing CBF changes in response to CPP and MAP variations. Several tools, either static or dynamic, are available to indirectly assess cerebrovascular autoregulation with minimal invasiveness. Pressure reactivity index (PRx), a parameter derived from ABP and ICP waveforms, is the most accepted continuous bedside cerebrovascular autoregulation assessment method, although its calculation may be affected by several artefacts.^82,83^ In practice, a PRx value below zero reflects an intact autoregulatory reserve. On the contrary, when PRx shifts from negative to positive values (PRx >0.3) impaired cerebrovascular reactivity can be suspected.^84,85^ Moreover, PRx plotted against CPP shows a U-shaped curve where the lowest point corresponds to the optimal CPP (CPP_opt_). This value seems to be associated with the best individual state of autoregulation and could potentially lead to a CPP_opt_-based therapy.^86,87^ The identification of CPP_opt_, and its re-assessment throughout the treatment course, is a promising tool that may lead to an individualisation of pressure ranges, which seems to be critical for TBI patient outcomes.^86^

## Fluid management: neutral versus positive balance

Fluid therapy is a cornerstone of the haemodynamic management of patients who have sustained moderate-to-severe TBI.^88^ Intravenous fluid administration aims to support CBF and cerebral oxygenation and minimise secondary brain injury.^89^ However, no strong recommendations on optimal fluid administration are defined.^89^ A recent consensus of the ESICM suggests avoiding restrictive fluid strategies but is based on weak evidence.^90^ Fluid infusions can have both favourable and unfavourable consequences. Liberal fluid administration may lead to cerebral oedema and intracranial hypertension (IHT) because of increased BBB permeability. On the other hand, permissive hypotension, tolerated in haemorrhagic trauma, may be detrimental in TBI patients.^91,92^ Recently, a sub-analysis of the CENTER-TBI study demonstrated that a positive fluid balance over the complete ICU stay is associated with worse outcomes in TBI patients. However, this is an observational study, and no randomised controlled trials have been performed on this topic.^92^

## Which fluid is best in traumatic brain injury?

Fluid therapy is extensively employed in neurointensive care in order to replace blood loss, third space fluid shifts and to counteract secondary injury. It is of note that both the type and tonicity of fluids administered significantly affect outcomes in TBI patients.^89^ At present, however, there is no high-quality evidence about the best choice of fluid, and current clinical practice varies considerably.^93,94^ The ESICM consensus on fluid management in acute brain-injured patients arrived at recommendations for the use of crystalloids for both maintenance (strong) and resuscitation (weak).^90^

Crystalloid-based therapy is the strategy of choice in trauma protocols, especially in a prehospital setting; glucose-containing solutions and hypotonic fluids may exert detrimental effects by increasing cerebral oedema and ICP, and are not recommended in TBI management. On the contrary, hypertonic solutions have been recommended for increasing plasma tonicity and decreasing cerebral oedema, although not as fluids for resuscitation but to treat IHT. Hence, fluid resuscitation in TBI patients should be managed maintaining physiological plasma tonicity by using isotonic fluids. On the other hand, colloid-based resuscitation strategies have been employed in the past with the aim of sustaining oncotic pressure, avoiding interstitial extravasation.^90,92,95^ However, the Saline versus Albumin Fluid Evaluation (SAFE) randomised trial, reported an increase in mortality rate and adverse neurological outcome when albumin was used compared with 0.9% saline.^96^ As stated in the more recent SAFE-TBI trial,^97^ this harmful effect seems to be related to ICP increases,^96,98^ probably because of the low osmolality of the albumin preparation used and the injured BBB integrity,^99,100^ thus leading to the shift of fluid into the brain.

## Conclusion

**Fig. 4:**
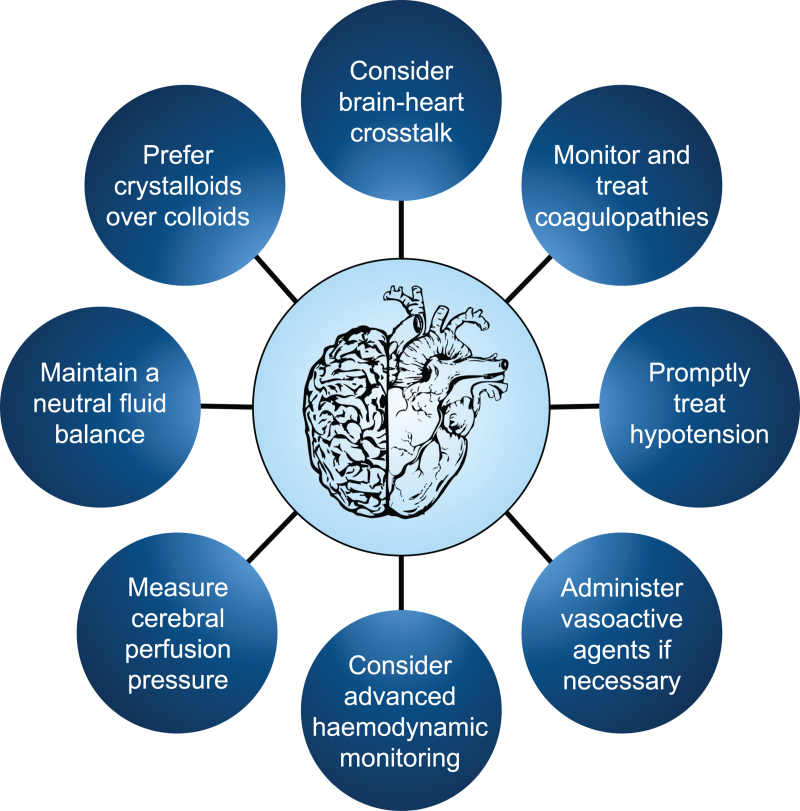
Eight simple rules for the thorough haemodynamic management of TBI adult patients, with the key points discussed in this review.

TBI is one of the leading causes of death and disability worldwide, and acute cardiac dysfunction and coagulopathy arising after TBI may further worsen the clinical picture.^34,101^ In this scenario, rigorous haemodynamic management, including advanced bedside monitoring and judicious fluid and drugs administration, and continuous neuromonitoring are of paramount importance in order to minimise the detrimental effects of secondary brain insult.^102^ Although some major progress has been achieved in the last decades, there remains a paucity of high-quality recommendations on the haemodynamic management of this population; thus further effort in performing research on this topic is required. In conclusion, haemodynamic management after TBI remains highly heterogeneous across various centres and largely derived from BTF guidelines. Comprehensive intensive care should involve a multidisciplinary team, and treatment protocols should be adjusted with the aim of reducing trauma-related disabling sequelae (Fig. 4).
